# Medium‐Entropy Monosilicates Deliver High Corrosion Resistance to Calcium‐Magnesium Aluminosilicate Molten Salt

**DOI:** 10.1002/advs.202400736

**Published:** 2024-04-19

**Authors:** Zeyu Chen, Yongzhe Wang, Yiling Huang, Fan Peng, Chucheng Lin, Wei Zheng, Xuemei Song, Yaran Niu, Yi Zeng

**Affiliations:** ^1^ The State Key Lab of High Performance Ceramics and Superfine Microstructure Shanghai Institute of Ceramics Chinese Academy of Sciences Shanghai 200050 China; ^2^ Center of Materials Science and Optoelectronics Engineering University of Chinese Academy of Sciences Beijing 100049 China; ^3^ Key Laboratory of Inorganic Coating Materials CAS Shanghai Institute of Ceramics Chinese Academy of Sciences Shanghai 200050 China

**Keywords:** CMAS corrosion, elemental diffusion, environmental barrier coatings, growth orientation of apatite, medium‐entropy monosilicates, surface energy

## Abstract

For decreasing the global cost of corrosion, it is essential to understand the intricate mechanisms of corrosion and enhance the corrosion resistance of materials. However, the ambiguity surrounding the dominant mechanism of calcium‐magnesium aluminosilicate (CMAS) molten salt corrosion in extreme environments hinders the mix‐and‐matching of the key rare earth elements for increasing the resistance of monosilicates against corrosion of CMAS. Herein, an approach based on correlated electron microscopy techniques combined with density functional theory calculations is presented to elucidate the complex interplay of competing mechanisms that control the corrosion of CMAS of monosilicates. These findings reveal a competition between thermodynamics and kinetics that relies on the temperatures and corrosion processes. Innovative medium‐entropy monosilicates with exceptional corrosion resistance even at 1500 °C are developed. This is achieved by precisely modulating the radii of rare earth ions in monosilicates to strike a delicate balance between the competition in thermodynamics and kinetics. After 50 and 100 h of corrosion, the thinnest reactive layers are measured to be only 28.8 and 35.4 µm, respectively.

## Graphical abstract

Innovative medium‐entropy monosilicates with exceptional corrosion resistance to CMAS are developed. This is achieved by precisely modulating the radii of RE^3+^ in monosilicates to strike a delicate balance between the competition in thermodynamics and kinetics. After 50 and 100 h of corrosion, the thinnest reactive layers are measured to be only 28.8 and 35.4 µm, respectively.

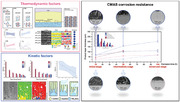

## 1 Introduction

The estimated cost of corrosion is 3–4% of a country's gross domestic product (GDP), reaching a global cost of $2.5 trillion in 2013.^[^ ^1^ ^]^ Therefore, reducing corrosion by exploiting corrosion‐resistant materials and gaining a deeper understanding of corrosion mechanisms hold economic incentives.^[^ ^2^ ^]^ The application of environmental barrier coatings (EBCs) on the surfaces of ceramic matrix composite (CMC) components used in gas‐turbine engines for aircraft propulsion and power generation is proving to be an effective strategy to combat high‐temperature corrosion.^[^ ^3^, ^4^, ^5^ ^]^

Silicate environmental deposits formed from runway debris, airborne sand, volcanic ash in aircraft engines, environmental dust, and fly ash in power generation engines are commonly known as calcium‐magnesium aluminosilicates (CMASs).^[^ ^6^, ^7^ ^]^ CMAS corrosion is a major threat that contributes to coating failure.^[^ ^8^, ^9^, ^10^ ^]^ The thickness of corrosion products serves as a measure of a material's resistance to CMAS corrosion, where CMAS does not penetrate the coating material, a thinner corrosion layer signifies reduced material consumption. Rare earth materials like zirconate,^[^ ^11^, ^12^, ^13^ ^]^ hafnate,^[^ ^14^, ^15^ ^]^ oxide,^[^ ^16^ ^]^ and tantalate^[^ ^17^ ^]^ exhibit superior performance as coating materials compared with traditional TBC materials, yttria‐stabilized zirconia (YSZ), at 1300–1350 °C. However, the surface temperature of EBCs may increase to ≥1400 °C.^[^ ^3^ ^]^ After exposure to corrosion at 1500 °C, the corrosion thickness of the novel RE_3_Al_5_O_12_/Al_2_O_3_ multiphase material^[^ ^18^, ^19^ ^]^ still exceeded 100 µm. Some studies have shown that rare earth pyrosilicates,^[^ ^20^ ^]^ especially high‐entropy pyrosilicates,^[^ ^3^, ^10^, ^21^ ^]^ with exceptional resistance to corrosive media under a service environment (>1400 °C), are promising candidates as EBC materials. However, a corrosion depth of <50 µm following long‐term exposure at 1500 °C remains a major challenge. At 1300 °C, rare earth monosilicates with small ionic radii of rare earth elements (REEs) exhibit excellent resistance to CMAS corrosion.^[^ ^22^ ^]^ Furthermore, the application of well‐considered doping strategies is able to increase the service temperature of monosilicates with excellent CMAS corrosion resistance up to 1500 °C.

The design strategies in the existing reports have not provided the desired level of corrosion resistance of CMAS. In many cases,^[^ ^23^, ^24^, ^25^ ^]^ the performance exhibited by multicomponent monosilicates during CMAS corrosion follows more of the mixing rule,^[^ ^26^ ^]^ i.e., the recession thicknesses of multicomponent monosilicates lie somewhere between those of single‐component monosilicates. Consequently, enhancing the CMAS corrosion resistance of monosilicates by using a rational mix‐and‐matching strategy remains challenging.

Effective sound design thinking mainly depends on thoughtful consideration of the following two pivotal issues:

(1) Thermodynamic factor

Since the melting points of most CMAS are in the range of 1200–1250 °C,^[^ ^27^, ^28^ ^]^ and EBCs are expected to operate at surface temperatures of ≥1400 °C,^[^ ^10^ ^]^ CMAS is prone to melt and adhere to the surface of EBCs in the form of glass deposits. The corrosion process primarily involves the EBC/CMAS reaction,^[^ ^29^ ^]^ and the nonstoichiometric Ca‐RE‐Si‐O apatite phase is a product of the reaction between monosilicates and CaO during monosilicate blocking CMAS corrosion.^[^ ^23^, ^30^ ^]^ The main corrosion reactions can be expressed using Equation (1)^[^ ^4^, ^6^, ^27^ ^]^:

(1) $$\begin{array}{ccc} & & 4\;RE_{2}SiO_{5}+2\;CaO\;\left(CMAS\right)+2\;SiO_{2}\;\left(CMAS\right)\\ & & =Ca_{2}\;RE_{8}{\left(SiO_{4}\right)}_{6}O_{2} \end{array}$$

Dissolution‐reprecipitation is a well‐documented mechanism for apatite product formation.^[^ ^31^, ^32^, ^33^ ^]^ Monosilicate particles wetted by molten CMAS dissolve to form REO_1.5_ and SiO_2_, which then diffuse into the CMAS. Ca‐RE‐Si‐O apatite crystals precipitate until the concentration of REO_1.5_ in CMAS reaches saturation. From the thermodynamic perspective, RE size can strongly affect the overall energetics of the apatite structure. The overall trend in the enthalpy of apatite formation with respect to the ionic potential suggests that the apatite structure becomes more stable as the ionic radius of RE^3+^ increases.^[^ ^34^ ^]^ Consequently, satisfying the thermodynamic conditions required for the formation of apatite from REEs with small ionic radii is more demanding.^[^ ^10^, ^23^, ^35^ ^]^ The recession layer thickness for RE_2_SiO_5_ (RE = Tb, Dy, Ho, Er, Y, Tm, Yb, and Lu) decreases almost linearly with the decreasing RE^3+^ ionic radius at 1300 °C.^[^ ^22^ ^]^

(2) Kinetic factor

However, it is important to note that all relevant corrosion processes (i.e., mainly dissolution, reprecipitation, and diffusion) are thermally activated, any variations in temperature and corrosion progress can cause changes in the dominant mechanism. The dynamic processes include the diffusion of elements within CMAS, apatite grains, monosilicate grains, and along grain boundaries. The next generation of EBCs will be subjected to harsh environments exceeding 1500 °C and the elemental diffusion accelerates with the increase in temperature. In addition, the dense layer of apatite effectively prevents the direct contact of CMAS with monosilicate with the increasing corrosion time. Subsequently, the movement of the apatite reaction front toward the interior of monosilicates, as a result of element diffusion with the increasing corrosion time, is chiefly responsible for the growth of apatite.^[^ ^29^ ^]^ Therefore, elemental diffusion mechanisms play an increasingly important role in the CMAS corrosion process.

REEs with small ionic radii can diffuse more readily, which exerts a negative effect at higher temperatures and during longer corrosion processes. REEs with various ionic radii exert opposing effects based on their thermodynamics and kinetics, and the interplay between thermodynamics and kinetics shapes the key features of the final corrosion outcomes. Therefore, achieving optimal corrosion resistance requires skillfully balancing adverse thermodynamic and kinetic effects through a rational mix‐and‐match strategy.

In the present investigation, to balance the thermodynamic and kinetic competition, the five REEs with the smallest ionic radius of the lanthanide series as well as the Y element with a similar ionic radius were methodically modulated. To this end, we start with Lu, which possesses the smallest ionic radius among the six REEs (to obtain a larger apatite formation enthalpy) and we progressively add elements with ionic radii close to Lu, which are supposed to gradually suppress elemental diffusion. Electron backscatter diffraction (EBSD) and transmission Kikuchi diffraction (TKD) techniques were utilized to determine the dominant orientations of apatite growth, influenced by various corrosion mechanisms and stages. Considering surface energy, elemental diffusion, apatite morphology, and growth strain, we examined the preferred orientations during the initial crystallization stage of apatite, as well as the evolution of orientation throughout the growth process. Therefore, this study lays a fairly solid foundation for the development of environmental barrier coatings that could be utilized in harsh environments and provides a compelling approach for the design of corrosion‐resistant materials.

## 2 Results

### 2.1 Phase Structure and Composition of the Uncorroded Monosilicate Blocks

**Figure 1:**
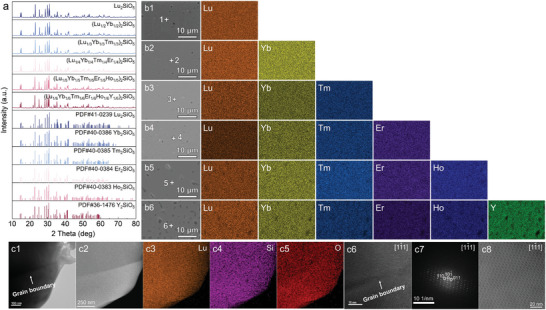
Phase structure and composition of six types of monosilicate blocks. a) XRD patterns of the six types of monosilicates, b) SEM images and EDS elemental mappings of the polished surface of the six monosilicate blocks, and c) TEM results of Lu_2_SiO_5_.

**Figure** **1**a illustrates the X‐ray diffraction (XRD) patterns of six distinct types of monosilicates, all of which exhibit the diffraction peaks of X2‐type monosilicates with space group of C_2_/c. The polished surface morphology and energy‐dispersive X‐ray spectroscopy (EDS) elemental mappings of six monosilicate blocks are presented in Figure 1b, where different REEs are evenly distributed and there is no micron‐scale elemental segregation. Supplementary material (Table S1, Supporting Information) presents the RE element contents at various points in Figure 1b, which correspond to the theoretical values. Taking Lu_2_SiO_5_ as an example, Figure 1c presents transmission electron microscopy (TEM) results for this monosilicate. The selected‐area electron diffraction (SAED) results also confirm its classification as an X‐2 type rare earth monosilicate with a space group of C_2_/c.

EBSD analysis was performed on the surface of all six types of monosilicate blocks to collect crystallographic information before the onset of corrosion; the results are illustrated in Figure S1 (Supporting Information). It is noteworthy that the monosilicates prepared through pressure‐less sintering did not exhibit a discernible crystal texture, as shown in Figure S1a2–f2 (Supporting Information). Arguably, the grain orientation of these underlying monosilicates plays a limited role during corrosion, given that their grains are equiaxed and randomly oriented. Figure S1a3–f3 (Supporting Information) shows images of local misorientation and quantitative statistics of local misorientation can be seen in Figure S1a6–f6 (Supporting Information). In addition, the grain size (see Figure S1a4–f4, Supporting Information) as well as the grain boundary angle (see Figure S1a5–f5, Supporting Information) may also partially affect the corrosion process, as the motion of atoms tends to be more active at the grain boundaries, but these characteristics of the six samples do not exhibit significant differences.

### 2.2 CMAS Corrosion Resistance and Corrosion Behavior at 1500 °C

**Figure 2:**
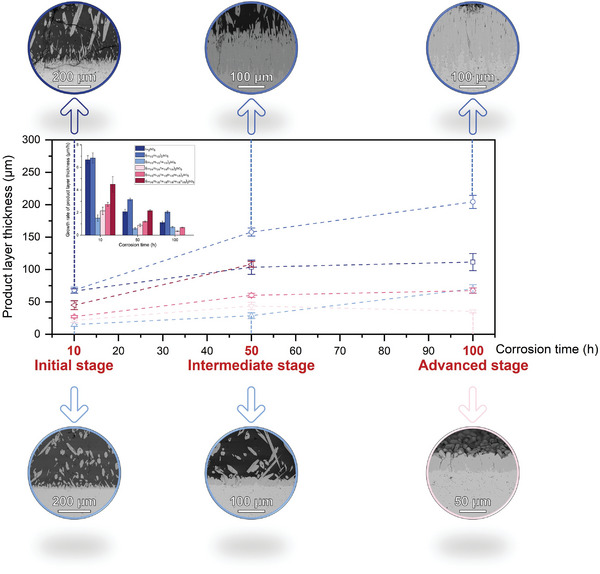
Product layer thickness and its growth rate at different stages of corrosion.

The service temperature of hot‐end components in new‐generation, high‐thrust‐to‐weight ratio aero‐engines can exceed 1400 °C, which is higher than the melting point of CMAS. Therefore, we evaluated the CMAS corrosion resistance of the six monosilicates at 1500 °C (for various durations, including 10, 50, and 100 h), which is closer to the service temperature of EBC materials. **Figure** **2** demonstrates the corrosion thicknesses of the six monosilicate blocks after corrosion for various durations. As the corrosion time increased from 10 h to 50 and 100 h, the average thickness of the corrosion layer continued to rise, ranging from 15.1–68.3 µm to 35.4–204.4 µm, while the growth rate notably decreased (from 1.51–6.83 to 0.35–2.04 µm h^−1^). Two medium‐entropy monosilicates—(Lu_1/3_Yb_1/3_Tm_1/3_)_2_SiO_5_ and (Lu_1/4_Yb_1/4_Tm_1/4_Er_1/4_)_2_SiO_5_ exhibited exceptional resistance to melt CMAS at service temperature. The lowest reactive layer thicknesses were only 28.8 and 35.4 µm after 50 and 100 h of corrosion at 1500 °C, which were only 27.8% and 31.7% of single‐component Lu_2_SiO_5_, respectively.

To date, this investigation has identified the thinnest corrosion layer observed at equivalent corrosion temperatures and durations based on our comprehensive literature review. Subsequently, a thorough investigation was conducted to understand the corrosion behavior and the potential mechanisms contributing to the changes in corrosion resistance.

#### 2.2.1 Initial Stage of Corrosion (10 h)

**Figure 3:**
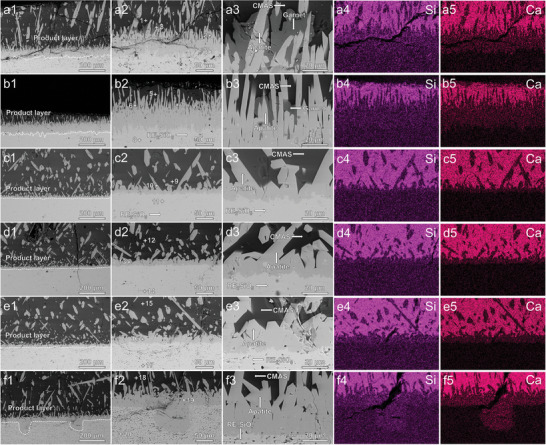
Microstructure and elemental composition of the six monosilicate blocks corroded by CMAS for 10 h. a) Lu_2_SiO_5_, b) (Lu_1/2_Yb_1/2_)_2_SiO_5_, c) (Lu_1/3_Yb_1/3_Tm_1/3_)_2_SiO_5_, d) (Lu_1/4_Yb_1/4_Tm_1/4_Er_1/4_)_2_SiO_5_, e) (Lu_1/5_Yb_1/5_Tm_1/5_Er_1/5_Ho_1/5_)_2_SiO_5_, and f) (Lu_1/6_Yb_1/6_Tm_1/6_Er_1/6_Ho_1/6_Y_1/6_)_2_SiO_5_.

XRD patterns of the six monosilicate blocks corroded by CMAS are shown in Figure S2 (Supporting Information). As shown, the diffraction peak on the block surface corresponded mainly to apatite (corrosion product), with some monosilicate peaks visible. **Figure** **3**a–f shows the cross‐sectional morphology and chemical element distribution of the six monosilicate blocks after corrosion at 1500 °C for 10 h. According to the EDS semi‐quantitative analysis of points 1–20 (Figure 3a2–f2) in the supplementary material (Table S2, Supporting Information), the substance (points 1, 5, 9, 12, 15, and 18) covering the top of the blocks with the darkest contrast was the residual CMAS, corresponding to the brightest parts of the Si and Ca elemental mappings. The substance (points 4, 8, 11, 14, 17, and 20) in the brightest contrast area at the bottom of the figure was uncorroded monosilicate. The grains (points 2, 6, 10, 13, 16, and 19) scattered in the CMAS melt or accumulated at the interface between the CMAS and the monosilicate blocks were the corrosion product apatite, corresponding to the secondary brightest part of the Ca elemental mappings. Additionally, a small number of grains (points 3 and 7) with a dark gray contrast corresponded to the garnet (corrosion product) of Lu_2_SiO_5_ and (Lu_1/2_Yb_1/2_)_2_SiO_5_. Garnet formation is a common phenomenon during CMAS corrosion of monosilicates, and as shown in Figure S3 (Supporting Information), garnets were detected on the surfaces of all six monosilicate blocks after corrosion.

Compared to the apatite dispersed in the CMAS melt, the apatite grains accumulated at the interface between CMAS and Lu_2_SiO_5_ and (Lu_1/2_Yb_1/2_)_2_SiO_5_ blocks have a more elongated shape. They are arranged regularly in a needle‐like direction to form a dense layer of corrosion products. However, apatite grains accumulated at the interfaces between CMAS and (Lu_1/3_Yb_1/3_Tm_1/3_)_2_SiO_5_, (Lu_1/4_Yb_1/4_Tm_1/4_Er_1/4_)_2_SiO_5_, (Lu_1/5_Yb_1/5_Tm_1/5_Er_1/5_Ho_1/5_)_2_SiO_5_, and (Lu_1/6_Yb_1/6_Tm_1/6_Er_1/6_Ho_1/6_Y_1/6_)_2_SiO_5_ blocks were coarserin shape. Lu_2_SiO_5_ and (Lu_1/2_Yb_1/2_)_2_SiO_5_ formed the thickest apatite corrosion layers with an average thickness of 66.8 and 68.3 µm, respectively. The average thicknesses of the corrosion product layer formed by these coarse apatite for the four monosilicates ((Lu_1/3_Yb_1/3_Tm_1/3_)_2_SiO_5_, (Lu_1/4_Yb_1/4_Tm_1/4_Er_1/4_)_2_SiO_5_, ((Lu_1/5_Yb_1/5_Tm_1/5_Er_1/5_Ho_1/5_)_2_SiO_5_, and (Lu_1/6_Yb_1/6_Tm_1/6_Er_1/6_Ho_1/6_Y_1/6_)_2_SiO_5_) in order were 15.0, 21.5, 27.3, and 45.0 µm, which became thicker with the growth of elemental species and much smaller than that of the needle‐like apatite layer formed by Lu_2_SiO_5_ and (Lu_1/2_Yb_1/2_)_2_SiO_5_.

#### 2.2.2 Intermediate Stage of Corrosion (50 h)

Figure S4a–f (Supporting Information) illustrates the corrosion of the six monosilicates at 1500 °C for 50 h. With the increase in the corrosion time to 50 h, the average thickness of the six monosilicate corrosion product layers increased to 103.5, 157.8, 28.8, 43.7, 60.2, and 108.0 µm, respectively. The thickness of both the corrosion product layer (comprising needle‐like apatite) and the corrosion product layer (consisting of coarse apatite) increased with an increase in the type of doping element. Similar to the case when the sample was corroded for 10 h, the dense layer comprising needle‐like apatite remained relatively thick, except that the corrosion product layer of (Lu_1/6_Yb_1/6_Tm_1/6_Er_1/6_Ho_1/6_Y_1/6_)_2_SiO_5_ measured 108.0 µm, slightly thicker than that of Lu_2_SiO_5_ (103.5 µm). However, no CMAS remained on the surface of the Lu_2_SiO_5_ block, while residual CMAS remained on the surface of the other five blocks, and this may be attributed to the highest diffusion rate of Lu.

#### 2.2.3 Advanced Stage of Corrosion (100 h)

When the corrosion time was increased to 100 h, the surfaces of all six monosilicate blocks were free of CMAS residues, and the corrosion became completed (see Figure S5a–f, Supporting Information). After 100 h of complete reaction, a large amount of apatite formed inside (Lu_1/6_Yb_1/6_Tm_1/6_Er_1/6_Ho_1/6_Y_1/6_)_2_SiO_5_ block, as well as a diffuse distribution of apatite with monosilicate inside the block were observed. This fact may be due to the fact that the presence of more pores in it provides more channels for CMAS penetration. However, this phenomenon did not occur in another highly porous sample, namely, (Lu_1/5_Yb_1/5_Tm_1/5_Er_1/5_Ho_1/5_)_2_SiO_5_. The average thickness of corrosion product layers of the other five monosilicates became 111.5, 204.4, 70.3, 35.4, and 67.4 µm, respectively. After 100 h of corrosion, the apatite thickness of (Lu_1/3_Yb_1/3_Tm_1/3_)_2_SiO_5_ was marginally larger at 70.3 µm compared to (Lu_1/4_Yb_1/4_Tm_1/4_Er_1/4_)_2_SiO_5_ (35.4 µm) and (Lu_1/5_Yb_1/5_Tm_1/5_Er_1/5_Ho_1/5_)_2_SiO_5_ (67.4 µm). (Lu_1/3_Yb_1/3_Tm_1/3_)_2_SiO_5_ also exhibited a corrosion layer with an irregular corrosion interface formed by downward apatite similar to Lu_2_SiO_5_ and (Lu_1/2_Yb_1/2_)_2_SiO_5_. This may be related to the alteration in the composition of CMAS after long‐term corrosion, promoting elemental diffusion during the corrosion process. The average thickness of the corrosion product layer of (Lu_1/4_Yb_1/4_Tm_1/4_Er_1/4_)_2_SiO_5_ corroded for 100 h (35.4 µm) was even less than that of (Lu_1/4_Yb_1/4_Tm_1/4_Er_1/4_)_2_SiO_5_ corroded for 50 h (43.7 µm). The reason may be as follows: after the sample was corroded for 50 h, a certain amount of CMAS remained on the surface, and during the cooling process, some apatite precipitated from the CMAS because of the contraction of the liquid phase field. However, after 100 h of heat preservation, the CMAS was completely volatilized, and thus, no apatite was produced during the cooling process.

The following sections provide a comprehensive exploration and discussion of the fundamental corrosion behavior and corrosion mechanisms, including the thermodynamically influenced nucleation mechanism of apatite and the kinetically influenced diffusion growth mechanism of apatite, all of which play a pivotal role in shaping the overall corrosion performance.

## 3 Discussion

### 3.1 Thermodynamic Factors

#### 3.1.1 Influence of Surface Energy and Enthalpy of Generation on Apatite Nucleation

**Figure 4:**
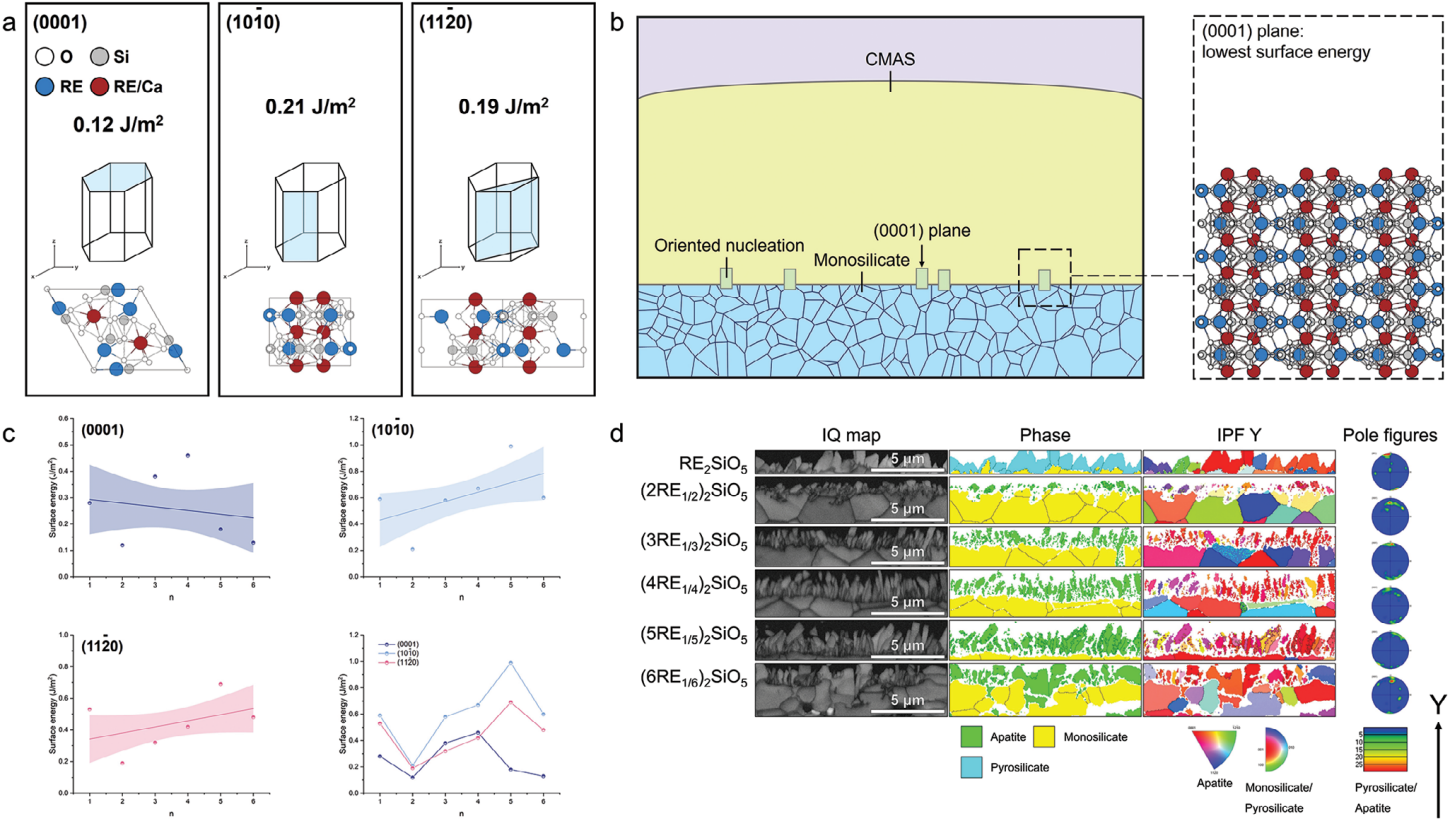
Nucleation mechanism of apatite during CMAS corrosion of monosilicates. a) Surface energy of three distinct apatite crystal faces. a) Surface energy of three distinct apatite crystal faces, b) Schematic illustration of the thermodynamic driving force of the oriented nucleation of apatite (0001) plane, c) Trends in three distinct surface energies of apatite products from six monosilicates, and d) EBSD results of the cross‐section of the six types of monosilicate corrode by CMAS at 1250 °C for 10 min.

The CMAS melt is a classical silicate melt, having a three‐dimensional bone‐like structure comprising tetrahedral [SiO_4_], with SiO_2_ and AlO_1.5_ serving as web formers.^[^ ^36^ ^]^ The melting point of the molten salt with the chemical formula Ca_33_Mg_10_Al_13_Si_44_ used in this corrosion investigation is 1223.2 °C.^[^ ^27^ ^]^ To investigate the nucleation mechanism of apatite products in the early stages of corrosion, determine the intrinsic activity of the reaction of monosilicates with CMAS, and eliminate the influence of external defects such as grain boundaries and porosity, we subjected the blocks to corrosion for 10 min at 1250 °C, which is slightly higher than the melting point of this CMAS. Using density functional theory (DFT) calculations and electron backscatter diffraction (EBSD), the influence of changes in the composition of REEs on apatite nucleation in the early stages of corrosion was examined. For the apatite product of (Lu_1/2_Yb_1/2_)_2_SiO_5_, the surface energies of the three main crystal faces ((0001), ($10\bar{1}0$), and ($11\bar{2}0$)) of hexagonal apatite calculated using the DFT were 0.12, 0.21, and 0.19 J m^−2^, respectively, with the densely stacked (0001) face showing the lowest surface energy (**Figure** **4**a). As shown in Figure 4c, the (0001) face consistently exhibited the significantly smallest or second smallest surface energy among several apatite crystal faces. Furthermore, the surface energy of the (0001) face exhibited a descending trend with the increase in REE species. The corrosion product apatite with the chemical formula Ca_2_RE_8_(SiO_4_)_6_O_2_ was classified as a defect‐free apatite.^[^ ^34^ ^]^ Four cationic sites (coordinated with nine oxygen ions) were jointly occupied by RE^3+^ and Ca^2+^, whereas the remaining six cationic sites (coordinated with seven oxygen ions) were occupied by the remaining RE^3+^ ions alone.^[^ ^34^ ^]^ The enthalpies of the formation of apatite containing different REE elements were found to differ. Specifically, the smaller the ionic radius of REE, the higher the enthalpy of apatite formation.^[^ ^34^ ^]^ As modeled in Figure 4b, this thermodynamic driving force led to the preferential formation of the (0001) orientation of apatite, which determined the crystallization texture and promoted the formation of apatite crystals with a wider variety of REEs with larger ionic radii. These observations were confirmed by the IPF Y images and the pole figure of the EBSD results shown in Figure 4d. The apatite products of the five types of monosilicate except Lu_2_SiO_5_ exhibited a (0001) texture along the Y‐axis. The orientations were referred to a coordinate system where the Y‐axis was perpendicular to the original monosilicate substrates. Notably, Lu_2_SiO_5_ did not produce apatite, but only intermediate pyrosilicate, which is probably due to the most difficult thermodynamic conditions for its apatite‐producing reaction. In addition, the thickness of the apatite layer, which indicated the extent of apatite formation, gradually increased with the increase in the number of REE species. The thickness of apatite layers formed by the other five monosilicates except Lu_2_SiO_5_ were 1.10, 1.22, 1.61, 1.78, and 1.84 µm, respectively. At this stage, freshly nucleated apatite grains were remarkably smaller than monosilicate grains, and apatite growth by penetration along grain boundaries and pores was less noticeable. Additionally, the direct contact between CMAS and monosilicate weakened the diffusion effect. It can be concluded that the variance in apatite nucleation at this point was solely attributed to a single thermodynamic factor.

#### 3.1.2 Nucleation Mechanism of Apatite

**Figure 5:**
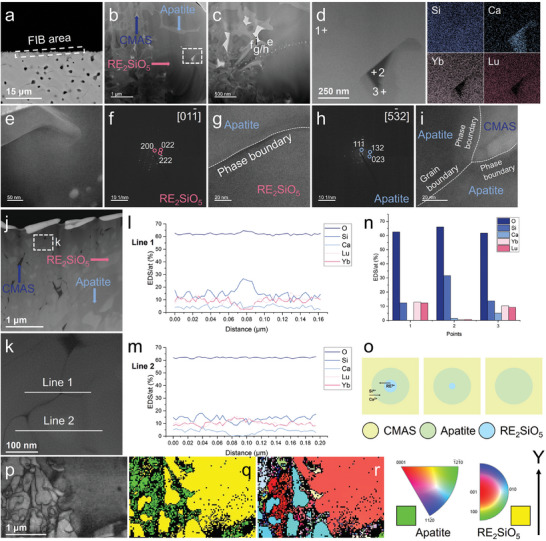
TEM and TKD results of (Lu_1/2_Yb_1/2_)_2_SiO_5_ corrode at 1250 °C for 10 min. a) TEM liftout area. a) TEM liftout area, b,c) Dark images, d) EDS elemental mappings, e,g,i) HRTEM images, f,h) SADE images, j,k) HAADF STEM micrograph, l,m) EDS line‐scan results, n) EDS semi‐quantitative analysis, o) The reaction model pertinent to the formation of the apatite by diffusion, p) Image quality map of TKD, q) Phase map of TKD, and r) IPF Y map of TKD.

Considering that the grain size (nanoscale) of some freshly nucleated apatite (especially that of (Lu_1/2_Yb_1/2_)_2_SiO_5_) was smaller than the spatial resolution of EBSD (0.1 µm), we sought to gain further insights into the mode of apatite nucleation during CMAS corrosion through identification of the corroded sample (Lu_1/2_Yb_1/2_)_2_SiO_5_ by using TEM combined with transmission Kikuchi diffraction (TKD). **Figure** **5**a illustrates the region of TEM liftouts under scanning electron microscopy (SEM). Figure 5b–n demonstrates the dark‐field images, HAADF STEM micrographs, SADE, and HRTEM images, as well as the EDS‐TEM results. By combining the elemental mapping results in Figure 5d, the EDS semi‐quantitative analysis results presented in Figure 5n (for substances with different contrasts given in Figure 5d), and the SADE images provided in Figure 5f,h, the substances with three different contrasts were identified, as shown in Figure 5b; these substances were CMAS, apatite, and monosilicate, in order. The clear phase boundaries between the apatite and monosilicate, as well as the grain boundaries between the apatite grains and the unclear phase boundaries between the apatite and CMAS, are depicted in Figure 5e,g,i. Throughout the TEM region of Figure 5b,j, the direct contact between the CMAS and monosilicates was minimal, and the apatite was always present as a transition substance between the CMAS and the monosilicates. As shown in Figure 5k and the EDS line‐scan results of Figure 5l, the amorphous CMAS also existed at the apatite grain boundaries. Many nanoscale monosilicate grains fully enclosed by apatite are visible in Figure 5b,j, and the compositional discrepancies between the monosilicate grains inside the apatite and the surrounding material can be observed in the EDS line‐scan results in Figure 5m. The TKD results in Figure 5p–r illustrate that the monosilicate grains (yellow phase) were surrounded by apatite (green phase) with sizes <1 µm on the left side of the phase map. In addition, these monosilicate grains exhibited the same orientation as shown in the IPF Y image, which suggests that these monosilicate grains belonged to the same intact grain before corrosion. Considering that the uncorroded monosilicate grains were mostly at the micrometer level, it can be assumed that the first step in the monosilicate grain corrosion by CMAS was to fragment the grains into numerous fine grains along the weaker parts inside the monosilicate grains and wrap them; then, apatite nuclei were formed at the interface between CMAS and the monosilicates, which led to the phenomenon of monosilicate wrapped by apatite, as shown in Figure 5o; then, the monosilicate was transformed into apatite through elemental diffusion. This transformation process was similar to the peritectic reaction of the metal. In this diffusion process, REEs diffused along the monosilicate–apatite–CMAS pathway according to the content gradient. The diffusion rates of various elements also exhibited variations in this procedure, and EDS semi‐quantitative analysis at point 2 in Figure 5n revealed that the elemental content of Lu in CMAS (0.56%) was slightly higher than that of Yb (0.44%), whereas reverse phenomenon was noted in apatite, with Lu content 9.18% being slightly lower than Yb content (10.29%), suggesting that the REEs with small ionic radii diffused faster in this pathway. The difference in the content of different REEs in CMAS versus apatite is a common phenomenon because of dissimilar ionic radii, as discussed in the next section.

### 3.2 Kinetic Factors

#### 3.2.1 Diffusion Rates of Various REEs

**Figure 6:**
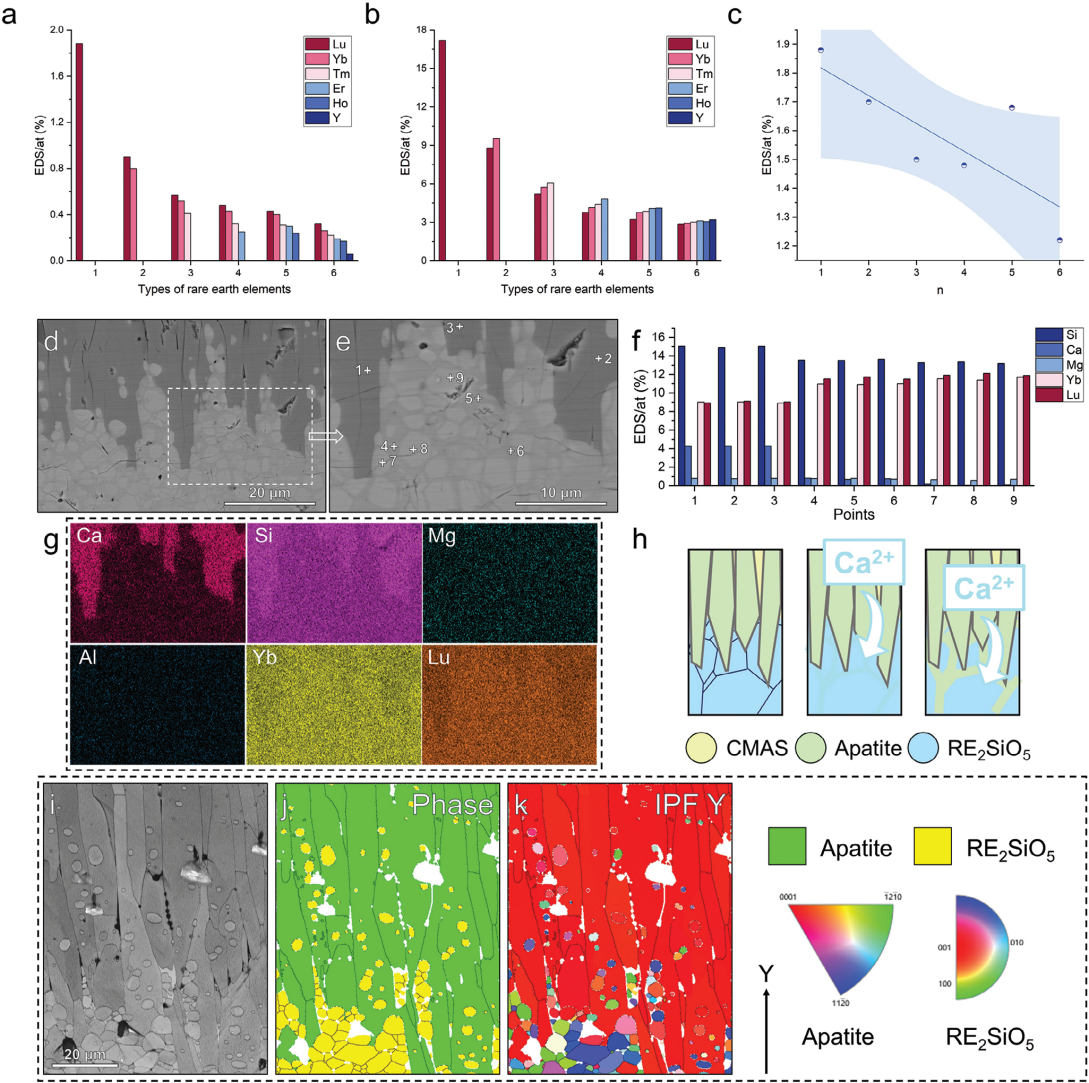
EDS semi‐quantitative analysis of six types of monosilicates after corrosion for 10 h, EDS and EBSD results of (Lu_1/2_Yb_1/2_)_2_SiO_5_ corroded by CMAS for 10 and 100 h. a) Content of six REEs in residual CMAS after corrosion for 10 h, b) Content of six REEs in apatite after corrosion for 10 h, c) Total REE contents in the residual CMAS after corrosion for 10 h, d,e) SEM images (10 h), f) EDS semi‐quantitative analysis (10 h), g) EDS elemental mappings (10 h), h) Diffusion growth modeling of apatite, i) Image quality map of EBSD (100 h), j) Phase map of EBSD (100 h), and k) IPF Y map of EBSD (100 h).

The difference between the results of long‐term corrosion at 1500 °C and the results of early stages of nucleation at 1250 °C may be because the apatite layer formed by long‐term corrosion blocked the direct contact of CMAS with the monosilicate blocks and high temperature enhanced the diffusion rate of elements; as a result, kinetics replaced thermodynamics as the dominant mechanism under these conditions. Discrepancies in the behaviors of multiple REEs in multi‐component monosilicates were also noted during the apatite precipitation. **Figure** **6**a,b illustrates the content of 1–6 different REEs in residual CMAS and apatite of six types of monosilicates after corrosion for 10 h, as measured by EDS. The content of RE elements in CMAS gradually decreased with an increase in the radius of RE^3+^; nevertheless, REE content in apatite gradually increased, indicating that REEs with smaller ionic radii diffused faster and were more likely to diffuse through the monosilicate–apatite–CMSA diffusion pathway into CMAS. A side‐by‐side comparison of the total REEs’ contents in the residual CMAS of the six samples is presented in Figure 6c; a descending trend was observed with the addition of REEs with larger ionic radii. The kinetic‐dominated diffusion corrosion mechanism resulted in the production of the thickest corrosion layers of Lu_2_SiO_5_ and (Lu_1/2_Yb_1/2_)_2_SiO_5_.

#### 3.2.2 Diffusion Growth Mechanism of Apatite

To further examine the CMAS corrosion mechanism related to elemental diffusion, we further analyzed the elemental composition under the apatite layer formed after 10 h of corrosion, taking the thickest corrosion layer of (Lu_1/2_Yb_1/2_)_2_SiO_5_ as an example. As shown in the backscattered electron micrograph in Figure 6d,e, monosilicate (points 4–9) was present in more than one contrast zone below the apatite (points 1, 2, and 3). The brighter‐contrast grains (points 7, 8, and 9) were surrounded by areas of darker contrast (points 4, 5, and 6). EDS elemental mapping could only detect the difference in elemental content between apatite and monosilicate, whereas detecting and distinguishing minor compositional differences in the monosilicate region was challenging. The EDS semi‐quantitative results of Figure 6e are presented in Figure 6f. The plotted results reveal that the contents of REEs at points 4, 5, and 6 were lower than those at points 7, 8, and 9, but the contents of Ca at points 4, 5, and 6 were higher than those at points 7, 8 and 9, reflecting the diffusion preference of Ca. In CMAS, Ca continuously diffuses from the apatite phase to the monosilicate phase through two pathways: bulk diffusion and grain boundary diffusion. The diffusion of elements into the monosilicate phase started at the grain boundaries, as shown in Figure 6h, as deduced from the results in Figure 6e,c. As the corrosion time progressed, the reaction fronts gradually moved from the grain boundary to the monosilicate interior until the apatite engulfed the entire monosilicate grain. When the corrosion is finished, it can be seen in the EBSD results of Figure 6i–k that there exist many fully encapsulated monosilicate grains that do not fully react inside the apatite grains, which represents the growth mechanism of apatite accomplished by elemental diffusion. The diffusion of Mg element is the fastest, followed by Ca elements, while the diffusion of Al elements was not observed. At temperatures above the CMAS melting point, the metal‐oxygen bonds in the melt are in equilibrium with continuous formation and breakage. Since Al─O and Si─O bonds are stronger and more difficult to break compared to Ca─O and Mg─O bonds, cations at network modifiers (i.e., Ca^2+^ and Mg^2+^) have much higher mobility than network formers (i.e., Si^4+^ and Al^3+^).^[^ ^37^ ^]^

#### 3.2.3 Factor for Apatite Orientation

Miraculously, the addition of Tm element alone resulted in a 78% reduction in the corrosion layer thickness of (Lu_1/3_Yb_1/3_Tm_1/3_)_2_SiO_5_ (15.0 µm) compared to (Lu_1/2_Yb_1/2_)_2_SiO_5_ (68.3 µm), completing the transformation of the thickness of the corrosion layer from the thickest to the thinnest after corrosion for 10 h. On the one hand, the addition of the Tm element inhibits the rapid diffusion rate of the Lu/Yb element so that (Lu_1/3_Yb_1/3_Tm_1/3_)_2_SiO_5_ exhibits a dissimilar corrosion phenomenon to the previous two cases. In addition, the difference in shape, size, and orientation of apatite caused by this transformation in turn affects the diffusion process.

**Figure 7:**
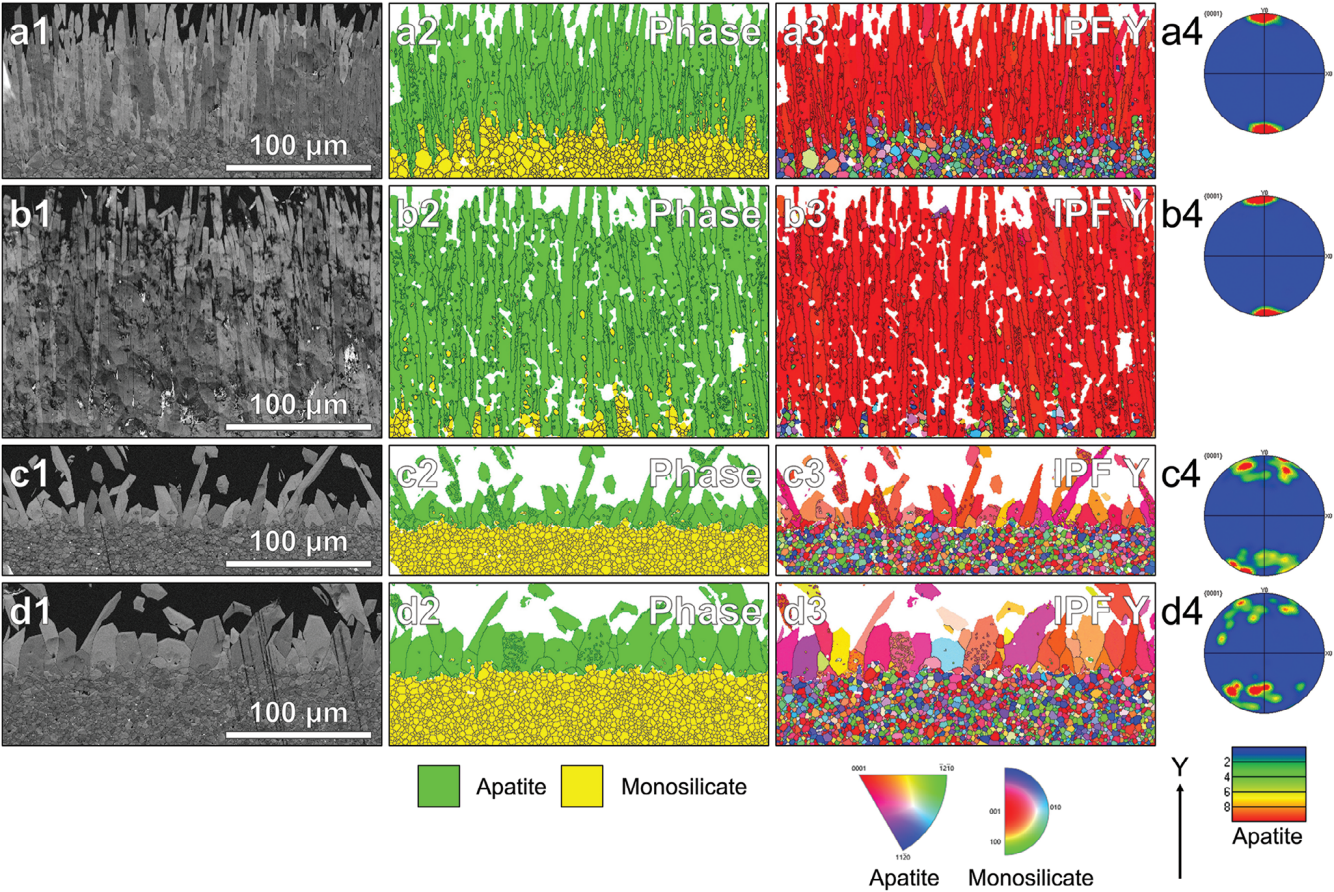
Typical EBSD datasets (image quality maps, phase maps, IPF Y maps, and pole figure of apatite). a) (Lu_1/2_Yb_1/2_)_2_SiO_5_ corroded for 10 h, b) (Lu_1/2_Yb_1/2_)_2_SiO_5_ corroded for 50 h, c) (Lu_1/3_Yb_1/3_Tm_1/3_)_2_SiO_5_ corroded for 10 h, and d) (Lu_1/3_Yb_1/3_Tm_1/3_)_2_SiO_5_ corroded for 50 h.

We further explored the differences and possible causes of apatite produced from (Lu_1/2_Yb_1/2_)_2_SiO_5_ and (Lu_1/3_Yb_1/3_Tm_1/3_)_2_SiO_5_ because of the addition of Tm, as determined by EBSD. **Figure** **7** displays the EBSD results of (Lu_1/2_Yb_1/2_)_2_SiO_5_ and (Lu_1/3_Yb_1/3_Tm_1/3_)_2_SiO_5_ after corrosion at 1500 °C for 10 and 50 h, respectively. The EBSD analysis revealed that the outer corrosion layer comprised oriented acicular apatite crystallites (green phase), while the inner monosilicate matrix (yellow phase) preserved the randomly oriented crystallographic information. The high‐intensity regions around *y*‐poles of the {0001} plot in Figure 7a indicate a preference in the orientation of the apatite c‐axis to the substrate normal. As presented in Figure 7b, this preference did not change as the corrosion time increased from 10 to 50 h. The thickness of the corrosion layer increased mainly due to the elongation of the long axis of apatite and the formation of new apatite grains. In contrast, the apatite product of (Lu_1/3_Yb_1/3_Tm_1/3_)_2_SiO_5_ had a weaker preference for the apatite c‐axis orientation, as shown in Figure 7c,d. As the corrosion time increased from 10 to 50 h, the growth of apatite size occurred due to lateral expansion. (Lu_1/2_Yb_1/2_)_2_SiO_5_ with more diffusible ions with smaller radii tended to grow diffusively down the concentration gradient as per the diffusion rule, as shown in Figure 6, and high diffusion rates were conducive to reaching the concentration saturation point required for nucleation. These factors jointly led to the production of elongated, needle‐like apatite. In turn, the presence of more grain boundaries in this apatite structure provided more channels for elemental diffusion.

When the corrosion time increased to 100 h, the CMAS was completely consumed via reaction, diffusion, and volatilization; as a result, no CMAS remained on the surface of any of the six monosilicate blocks, and the corrosion process stopped. The typical apatite characteristics of Figure 7 remained after corrosion for 100 h, as shown in Figure S6 (Supporting Information). Figure S6a (Supporting Information) shows the EBSD datasets of the six monosilicate blocks at the end of CMAS corrosion. As illustrated in Figure S6a,b (Supporting Information), only the crystal face (0001) of hexagonal apatite produced by Lu_2_SiO_5_ and (Lu_1/2_Yb_1/2_)_2_SiO_5_ tended to grow in the direction perpendicular to the interface between CMAS and monosilicate blocks (Y direction). Each apatite grain exhibited a fibrous texture in which the needles were misoriented relative to each other by rotation around the *c*‐axis. Some small unreacted monosilicate grains enclosed by apatite were observed inside some of the apatite grains. As mentioned earlier, the reason for the formation of this apatite texture, which was detrimental to the corrosion resistance of materials, may be related to the rapid diffusion of REE elements with small ionic radii and the diffusion growth mechanism of apatite.

The EBSD datasets of (Lu_1/3_Yb_1/3_Tm_1/3_)_2_SiO_5_, (Lu_1/4_Yb_1/4_Tm_1/4_Er_1/4_)_2_SiO_5_, and (Lu_1/5_Yb_1/5_Tm_1/5_Er_1/5_Ho_1/5_)_2_SiO_5_ corroded by CMAS are demonstrated in Figure S6c–e (Supporting Information). Distinguished from the acicular apatite of Lu_2_SiO_5_ and (Lu_1/2_Yb_1/2_)_2_SiO_5_, the corrosion products of these three monosilicates were coarse in shape. Moreover, the preferred orientation of apatite weakened with an increase in the elemental species. Fewer grain boundaries favored elemental diffusion in coarser apatite, which increased the corrosion resistance of the block. The apatite layer, which acted as a barrier, was replaced by penetrative corrosion of the entire block after (Lu_1/6_Yb_1/6_Tm_1/6_Er_1/6_Ho_1/6_Y_1/6_)_2_SiO_5_ was corroded for 100 h. This can be attributed to the lower thermodynamic energy of (Lu_1/6_Yb_1/6_Tm_1/6_Er_1/6_Ho_1/6_Y_1/6_)_2_SiO_5_ to form apatite and higher porosity of the block. In addition, the orientation of the corrosion products (Lu_1/6_Yb_1/6_Tm_1/6_Er_1/6_Ho_1/6_Y_1/6_)_2_SiO_5_ was completely random along the y‐axis.

The pole figure obtained from the apatite product or monosilicate substrate is illustrated in Figure S6a5–f5 and a6–f6 (Supporting Information). As demonstrated in Figure S6a5 and b5 (Supporting Information), two isolated groups of hexagonal grains can generally be found in the {0001} pole figure. As the type of monosilicate element increases from 2 to 6, the orientation of its product apatite is gradually dispersed, until it shows no crystal texture. However, the remaining six monosilicates after corrosion all retained the information of randomly oriented crystallography (Figure S6a6–f6, Supporting Information).

#### 3.2.4 Factor for Apatite Growth Strain

Another factor that contributed to the morphological differences in apatite was the volume change associated with the monosilicate‐to‐apatite transformation. The protective product layer required a ratio greater than one, that is, the volume of the elementary cell of the corrosion product was larger than that of the corresponding coating material.^[^ ^2^ ^]^ As an example, the ratio between Ca_2_Lu_8_(SiO_4_)_6_O_2_ and Lu_2_SiO_5_ was 1.2. The build‐up of compressive stresses, which impact apatite grain growth, and thereby grain nucleation, was associated with the volume expansion of a protective product layer. This was because the apatite grew to minimize its biaxial or triaxial stress state. Considering the effect of volume change along with orientation and elastic anisotropy, apatite may preferentially grow in a particular shape and orientation, which maximizes the accommodation of volume changes normal to the interface.

**Figure 8:**
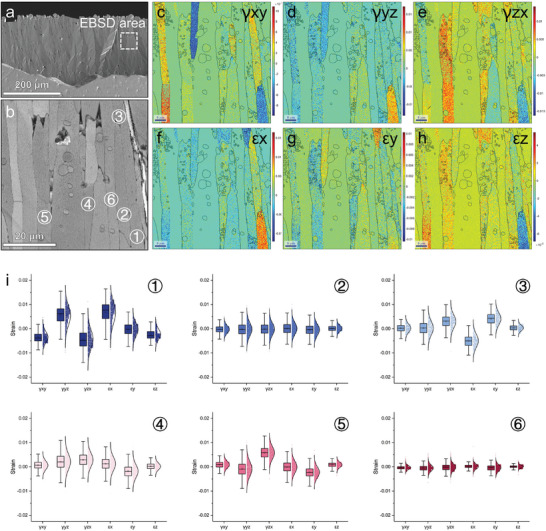
The strain growth distribution and quantitative analysis of the final apatite products of (Lu_1/2_Yb_1/2_)_2_SiO_5_. a,b) SEM images, c–h) The strain growth distribution, and i) Quantitative analysis.

The strain growth distribution and quantitative analysis of the final apatite products of (Lu_1/2_Yb_1/2_)_2_SiO_5_ and (Lu_1/3_Yb_1/3_Tm_1/3_)_2_SiO_5_ (**Figure** **8**; Figure S7, Supporting Information) were determined using EBSD patterns. Irrespective of whether the strain was shear or linear, the strain distribution differed markedly between the acicular apatite grains of (Lu_1/2_Yb_1/2_)_2_SiO_5_. Considering the linear strain in the y‐direction as an example, grain 3 exhibited positive strain, which was the driving force for the downward growth of apatite. In contrast, grain 5 showed negative strain, which may be caused by compression during apatite growth. The growth of apatite also led to strain release (grains 1, 2, 4, and 6). However, the stress distribution in the apatite grains of (Lu_1/3_Yb_1/3_Tm_1/3_)_2_SiO_5_ (Figure S7, Supporting Information) was relatively uniform and constant, which may be attributed to the strain release with lateral growth of the grains, which in turn weakened the driving force for the downward growth of apatite. The interplay between a diffusion‐dominated apatite growth mechanism and the inherent heterogeneous growth strain of apatite culminated in the formation of needle‐like apatite structures with specific orientations that exhibited poor corrosion resistance.

**Figure 9:**
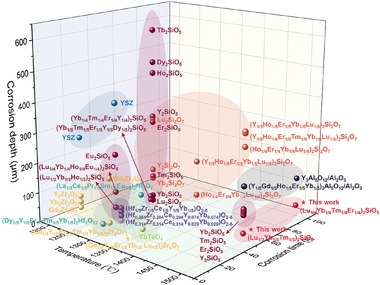
Summary of recently reported representative data, along with data from this study, regarding the corrosion depth of EBC/TBC materials when exposed to molten CMAS.^[^ ^3^, ^10^, ^11^, ^12^, ^13^, ^14^, ^15^, ^16^, ^17^, ^18^, ^19^, ^20^, ^22^, ^24^, ^25^, ^38^, ^39^, ^40^ ^]^

Kinetic factors distinguish six monosilicates were divided into two groups based on the corrosion mechanism at 1500 °C: Lu_2_SiO_5_ and (Lu_1/2_Yb_1/2_)_2_SiO_5_ with similar acicular apatite products and (Lu_1/3_Yb_1/3_Tm_1/3_)_2_SiO_5_, (Lu_1/4_Yb_1/4_Tm_1/4_Er_1/4_)_2_SiO_5_, (Lu_1/5_Yb_1/5_Tm_1/5_Er_1/5_Ho_1/5_)_2_SiO_5_, and (Lu_1/6_Yb_1/6_Tm_1/6_Er_1/6_Ho_1/6_Y_1/6_)_2_SiO_5_ with coarse apatite. Monosilicates belonging to the same group with similar corrosion products also obeyed the thermodynamic laws. It can be said that kinetic factors played a major role, while thermodynamic factors played a very crucial but secondary role. Balancing the competition between thermodynamics and kinetics bestowed (Lu_1/3_Yb_1/3_Tm_1/3_)_2_SiO_5_ (50 h) and (Lu_1/4_Yb_1/4_Tm_1/4_Er_1/4_)_2_SiO_5_ (100 h) with the best corrosion resistance. The corrosion layers on these materials measured merely 28.8 µm after 50 h and 35.4 µm after 100 h at 1500 °C. According to available literature,^[^ ^3^, ^10^, ^11^, ^12^, ^13^, ^14^, ^15^, ^16^, ^17^, ^18^, ^19^, ^20^, ^22^, ^24^, ^25^, ^38^, ^39^, ^40^ ^]^ these medium‐entropy silicates show the slimmest corrosion thickness among comparable T/EBC materials under similar corrosion conditions, as demonstrated in **Figure** **9**. The specific power output of a gas turbine engine increases proportionally with the inlet temperature. To ensure safe operation, temperatures are typically restricted to 1200–1250 °C using thermal barrier coatings and cooling systems. However, the hot end components of newer aero‐engines with high thrust‐to‐weight ratios often exceed 1400 °C. Ceramic matrix composites are being considered to replace high‐temperature alloys for even higher temperatures. Nonetheless, using CMC requires environmental barrier coatings for protection. As service temperatures rise, EBC will encounter increasingly severe corrosion challenges. The availability of two medium‐entropy monosilicates offers the potential to develop EBC suitable for service at 1500 °C. This could lead to significant economic benefits by reducing corrosion‐related costs.

## 4 Conclusion

In this study, we investigated the CMAS corrosion resistance of six monosilicates. Thermodynamics predominantly influence the initial crystallization stage of the corrosion product, apatite. The primary reason for the preferential orientation of apatite is the low surface energy of the crystal surface (0001). However, as the temperature increases to the actual service temperature of 1500 °C, kinetics assume a more critical role in the growth of apatite. Morphological changes in apatite are attributed to growth strain. Two medium‐entropy monosilicates—(Lu_1/3_Yb_1/3_Tm_1/3_)_2_SiO_5_ and (Lu_1/4_Yb_1/4_Tm_1/4_Er_1/4_)_2_SiO_5_—designed using the rational mix‐and‐matching strategy demonstrated ideal resistance to melt CMAS at service temperature. The minimum thickness of the reactive layer was 28.8 and 35.4 µm obtained after 50 and 100 h of corrosion at 1500 °C, respectively. Our results confirmed that component tuning methods to balance competing thermodynamics and kinetics could be an effective means to enhance the resistance of materials to CMAS corrosion.

## 5 Experimental Section

### Sample Preparation

Six types of monosilicates (Lu_2_SiO_5_, (Lu_1/2_Yb_1/2_)_2_SiO_5_, (Lu_1/3_Yb_1/3_Tm_1/3_)_2_SiO_5_, (Lu_1/4_Yb_1/4_Tm_1/4_Er_1/4_)_2_SiO_5_, (Lu_1/5_Yb_1/5_Tm_1/5_Er_1/5_Ho_1/5_)_2_SiO_5_, and (Lu_1/6_Yb_1/6_Tm_1/6_Er_1/6_Ho_1/6_Y_1/6_)_2_SiO_5_) were prepared by a solid‐phase method. The powders of RE_2_O_3_ (RE = Lu, Yb, Tm, Er, Ho, and Y) and SiO_2_ were used as the starting materials. The mole ratio of each RE_2_O_3_ was equal, and the mole ratios of the total RE_2_O_3_ to SiO_2_ were 1:1. The powder container was homogeneously mixed in a vertical nylon tank by a ball mill using ethanol and zirconia balls as the dispersion medium. The resulting slurry was dried at 120 °C for 5 h and then passed through a sieve. The sieved powders were placed into circular molds with diameters of 15 and 20 mm, respectively, and cold‐pressed for 20 s at a pressure of 200 MPa. After cold pressing, the green billets were placed in a muffle furnace and held at 1500 °C for 5 h and 1700 °C for 10 h to obtain different ceramic blocks. CMAS powders were prepared using the solid‐phase method, with a molar ratio of Ca_33_Mg_10_Al_13_Si_44_. This involved utilizing CaO, MgO, Al_2_O_3_, and SiO_2_ as starting materials. Following the stoichiometric ratio, the mixture was thoroughly blended in a vertical nylon tank via ball milling for 4 h. The resulting slurry was then dried at 120 °C and screened through a 20‐mesh sieve. The mixed powders were subjected to a temperature of 1400 °C for 4 h in a muffle furnace, followed by cooling to 800 °C at a rate of 10 °C min^−1^. Subsequently, they were allowed to cool to room temperature, resulting in the formation of a CMAS block. This block was subsequently ground to obtain CMAS powder.

### CMAS Corrosion Tests

The milled CMAS powder was blended with ethyl alcohol and evenly applied onto the block surfaces. To ensure consistency, the CMAS concentration was adjusted to ≈35 mg cm^−2^ through successive coating and drying cycles. Subsequently, the blocks coated with CMAS powders were transferred to a muffle furnace and maintained in an air atmosphere at 1250 °C for 10 min and at 1500 °C for 10, 50, and 100 h, respectively.

### Structure and Compositional Characterization

The cross‐section of corroded samples and the surface of uncorroded samples were first mechanically ground and polished down to a 2500‐grit size. Second, the ion‐beam polishing was carried out with the Leica EM TIC 3X for 45 min to remove the strained layer of the surface, which might be caused by mechanical polishing. A scanning electron microscope (SEM, Magellan 400, FEI; USA) equipped with an energy‐dispersive X‐ray spectrometer (EDS; Oxford, England) and an electron backscatter diffraction (EBSD, Home developed) attachment was used to characterize the microscopic morphology, elemental composition and orientation information of the surface or cross‐section of the corroded monosilicate blocks. The polished surfaces of the uncorroded sample were also characterized by the same Scanning Electron Microscope System. In addition, the quantitative strain tensor distribution was evaluated by an in‐house coded high‐resolution EBSD (HREBSD) method (LRR‐HREBSD software), where the strain tensors were mainly derived from the shifts of the zone axis in the high‐quality Kikuchi patterns by digital image correlation (DIC). The arbitrary vector r shifts to r’ by displacement gradient tensor, where u_1_, u_2_, and u_3_ are the displacements along the x_1_, x_2_, and x_3_ axes, respectively. A more detailed algorithm could be referred to in the previous article.^[^ ^41^ ^]^

(2) $$r^{\prime }=r*\left[\begin{array}{ccc} \frac{\partial u1}{\partial x1} & \frac{\partial u1}{\partial x2} & \frac{\partial u1}{\partial x3}\\ \frac{\partial u2}{\partial x1} & \frac{\partial u2}{\partial x2} & \frac{\partial u2}{\partial x3}\\ \frac{\partial u3}{\partial x1} & \frac{\partial u3}{\partial x2} & \frac{\partial u3}{\partial x3} \end{array}\right]$$

The thickness of the corrosion product layer is determined by the average of five data. A TEM liftout was made from the position shown in Figure 5a of the prepared corrode cross‐section using the in situ liftout technique with an FEI focused‐ion beam (FIB) instrument. These samples were thinned to electron transparency using the standard FIB procedure with a final low‐energy cleaning step. Transmission electron microscopy (TEM, HF5000, Hitachi, Japan) and energy dispersive X‐ray spectrometry (EDS, Oxford, England) were used to characterize the microstructure and composition uniformity of the Lu_2_SiO_5_ blocks and the FIB sample. Phase composition analysis of the six types of blocks corroded by CMAS was conducted using an X‐ray diffraction method (XRD, D8 ADVANCE, Bruker, Germany). The X‐ray power settings were 40 kV and 40 mA, with data collection performed using a 0.6 mm evanescent slit. Data were recorded over a 2θ range of 10°–110° with a scanning rate of 0.8° min^−1^, and a range of 10°–80° with a scanning rate of 10° min^−1^.

### Surface Energy Calculations

Using the Full‐prof program, the structure information of the six apatite products was obtained by the Rietveld refinement method, as shown in Figure S8 and Table S3 (Supporting Information). All calculations were conducted using the Materials Studio (MS) in the Cambridge Sequential Total Energy Package (CASTEP) module. The generalized gradient approximation (GGA) functional by the Perdew–Burke–Ernzerhof (PBE) was employed. The cutoff energy was 489.8 eV, the pseudopotentials were OTFG ultrasoft, and the corresponding k‐point sampling was 2 × 2 × 1 for geometry optimization. The Broyden–Fletcher–Goldfarb–Shanno (BFGS) scheme was selected as the minimization algorithm. A vacuum layer was set 15 Å along the Z‐axis. The convergence tolerance of energy was 2 × 10^−5^ eV per atom, the maximum force was 0.05 eV Å^−1^, the maximum stress was 0.1 Gpa, and the maximum displacement was 0.002 Å.

The surface energy of the crystal surface was calculated according to the following equation:

(3) $$\;E_{\mathrm{sur}}=\;\left(E_{\mathrm{rel}}-nE_{\mathrm{bul}}\right)/2S$$

Where *E*_rel_ is the energy of the relaxed slab, *E*_bul_ is the energy of the bulk unit cell, *S* is the surface area of the slab, and *n* is the number of bulk unit cells required to form these slabs.

## Conflict of Interest

The authors declare no conflict of interest.

## Supporting information

Supporting Information

## Data Availability

The data that support the findings of this study are available from the corresponding author upon reasonable request.
