# Supplementary material for: Medium‐Entropy Monosilicates Deliver High Corrosion Resistance to Calcium‐Magnesium Aluminosilicate Molten Salt
Source: Adv Sci (Weinh). 2024 Apr 19;11(28):2400736. doi: 10.1002/advs.202400736 (PMC11267391; doi:10.1002/advs.202400736)
Supplement: Supplementary file 1 — Supporting Information [file ADVS-11-2400736-s001.pdf]

ADVANCED SCIENCE
Open Access

Supporting Information

for *Adv. Sci.*, DOI 10.1002/advs.202400736

Medium-Entropy Monosilicates Deliver High Corrosion Resistance to Calcium-Magnesium Aluminosilicate Molten Salt

Zeyu Chen, Yongzhe Wang, Yiling Huang, Fan Peng, Chucheng Lin, Wei Zheng, Xuemei Song, Yaran Niu and Yi Zeng**

Supporting Information

Title Medium-entropy monosilicates deliver high corrosion resistance to calcium-magnesium aluminosilicate molten salt

*Author(s), and Corresponding Author(s)**

Zeyu Chen, Yongzhe Wang, Yiling Huang, Fan Peng, Chucheng Lin, Wei Zheng, Xuemei Song, Yaran Niu, Yi Zeng**

A *Zeyu Chen, Yongzhe Wang*, Yiling Huang, Fan Peng, Chucheng Lin, Wei Zheng, Xuemei Song, Yi Zeng**

B *Zeyu Chen*

C *Yaran Niu*

A The State Key Lab of High Performance Ceramics and Superfine Microstructure, Shanghai Institute of Ceramics, Chinese Academy of Sciences, Shanghai, 200050, China.

B Center of Materials Science and Optoelectronics Engineering, University of Chinese Academy of Sciences, Beijing, 100049, China.

C Key Laboratory of Inorganic Coating Materials CAS, Shanghai Institute of Ceramics, Chinese Academy of Sciences, Shanghai, 200050, China.

E-mail: zengyi@mail.sic.ac.cn (Yi Zeng); wangyongzhe@mail.sic.ac.cn (Yongzhe Wang)

Figure S1.

Typical EBSD datasets of the uncorroded monosilicate surfaces. a Lu_2SiO_5, b $(Lu_{1/2}Yb_{1/2})_2SiO_5$, c $(Lu_{1/3}Yb_{1/3}Tm_{1/3})_2SiO_5$, d $(Lu_{1/4}Yb_{1/4}Tm_{1/4}Er_{1/4})_2SiO_5$, e $(Lu_{1/5}Yb_{1/5}Tm_{1/5}Er_{1/5}Ho_{1/5})_2SiO_5$, and f $(Lu_{1/6}Yb_{1/6}Tm_{1/6}Er_{1/6}Ho_{1/6}Y_{1/6})_2SiO_5$.

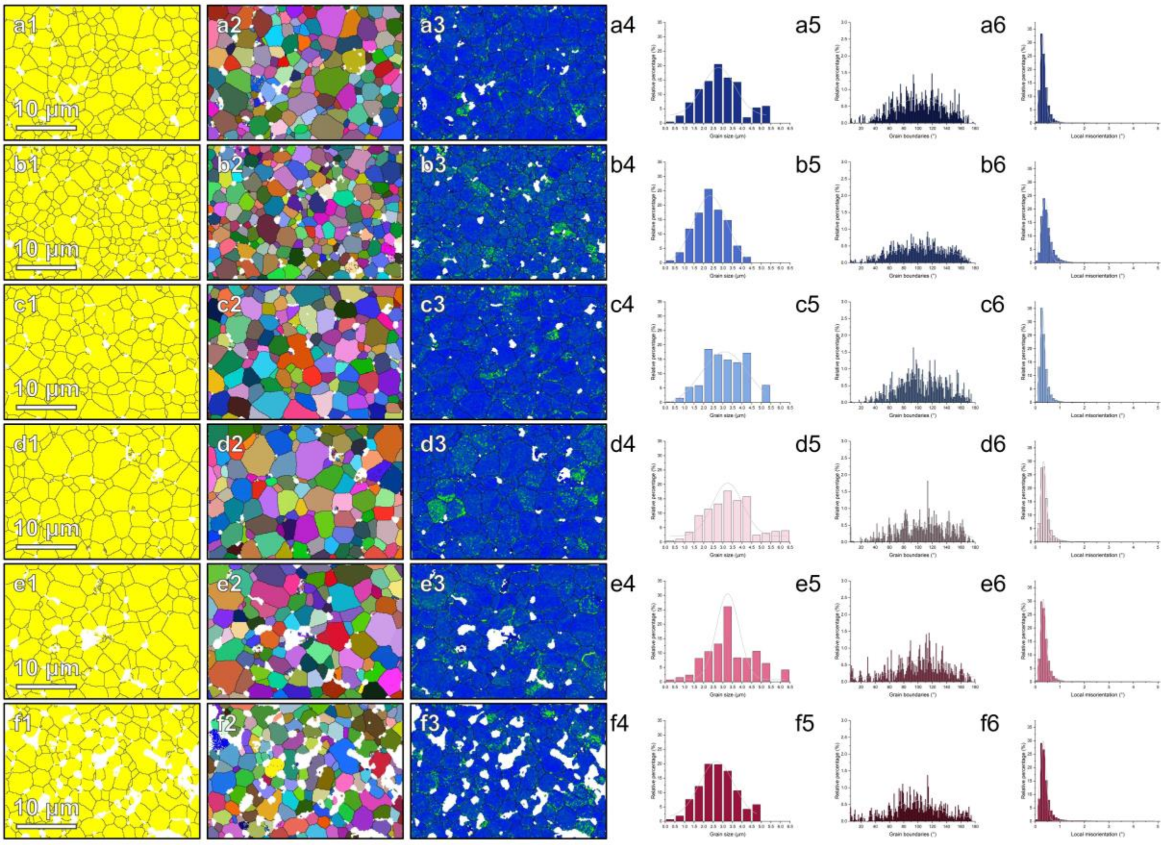

Figure S2.

XRD patterns of CMAS post-corroded surfaces of six samples are presented. a Lu_2SiO_5, b $(Lu_{1/2}Yb_{1/2})_2SiO_5$, c $(Lu_{1/3}Yb_{1/3}Tm_{1/3})_2SiO_5$, d $(Lu_{1/4}Yb_{1/4}Tm_{1/4}Er_{1/4})_2SiO_5$, e $(Lu_{1/5}Yb_{1/5}Tm_{1/5}Er_{1/5}Ho_{1/5})_2SiO_5$, and f $(Lu_{1/6}Yb_{1/6}Tm_{1/6}Er_{1/6}Ho_{1/6}Y_{1/6})_2SiO_5$.

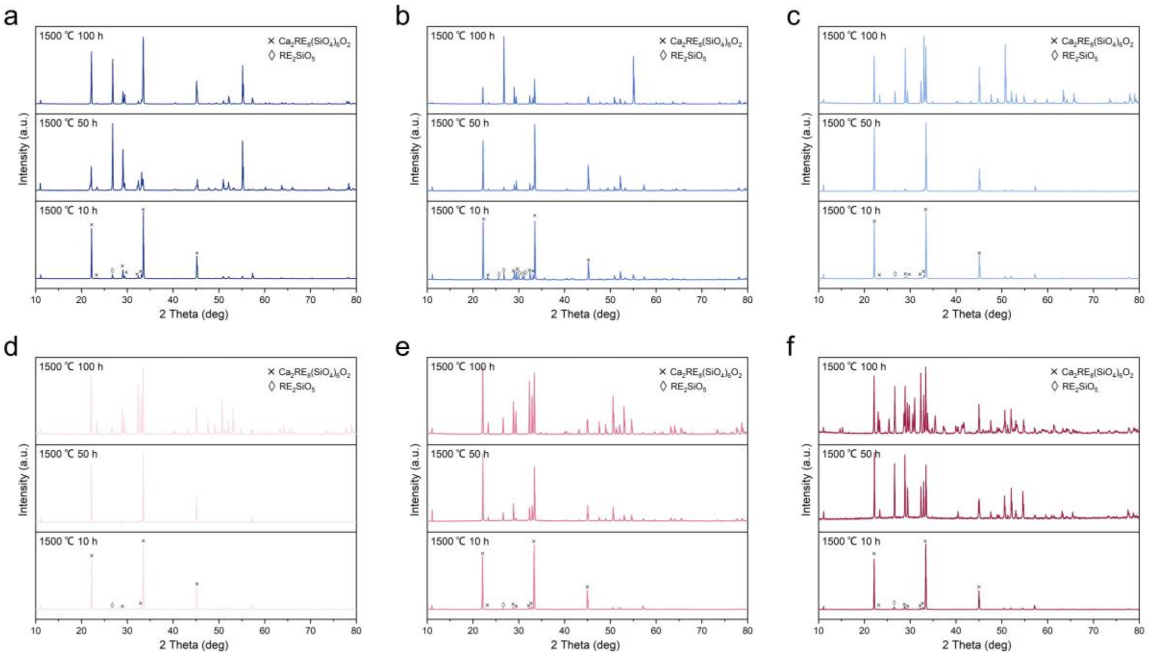

Figure S3.

CMAS post-corroded surfaces of six samples (10 h). a Lu_2SiO_5, b $(Lu_{1/2}Yb_{1/2})_2SiO_5$, c $(Lu_{1/3}Yb_{1/3}Tm_{1/3})_2SiO_5$, d $(Lu_{1/4}Yb_{1/4}Tm_{1/4}Er_{1/4})_2SiO_5$, e $(Lu_{1/5}Yb_{1/5}Tm_{1/5}Er_{1/5}Ho_{1/5})_2SiO_5$, and f $(Lu_{1/6}Yb_{1/6}Tm_{1/6}Er_{1/6}Ho_{1/6}Y_{1/6})_2SiO_5$.

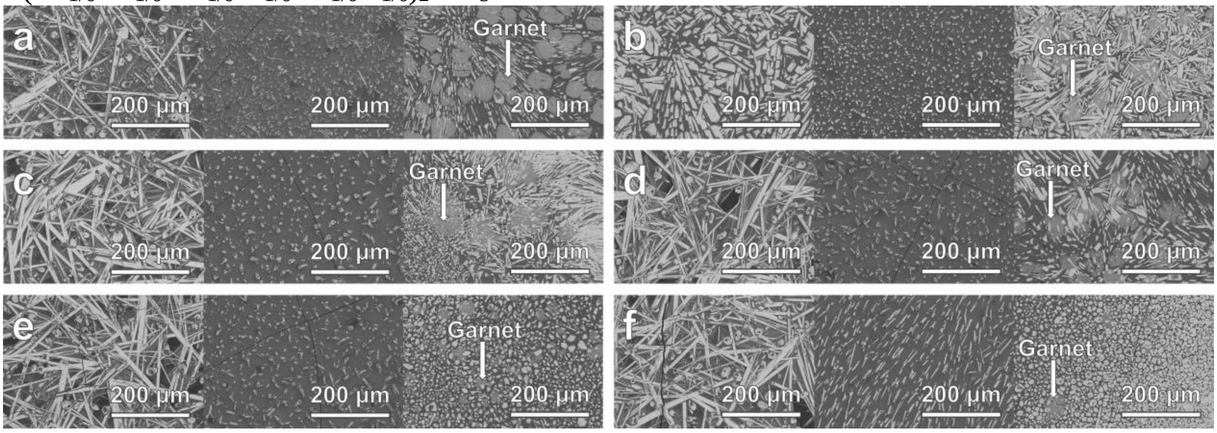

Figure S4.

CMAS post-corroded cross-sections of six samples (50 h). a Lu_2SiO_5, b $(Lu_{1/2}Yb_{1/2})_2SiO_5$, c $(Lu_{1/3}Yb_{1/3}Tm_{1/3})_2SiO_5$, d $(Lu_{1/4}Yb_{1/4}Tm_{1/4}Er_{1/4})_2SiO_5$, e $(Lu_{1/5}Yb_{1/5}Tm_{1/5}Er_{1/5}Ho_{1/5})_2SiO_5$, and f $(Lu_{1/6}Yb_{1/6}Tm_{1/6}Er_{1/6}Ho_{1/6}Y_{1/6})_2SiO_5$.

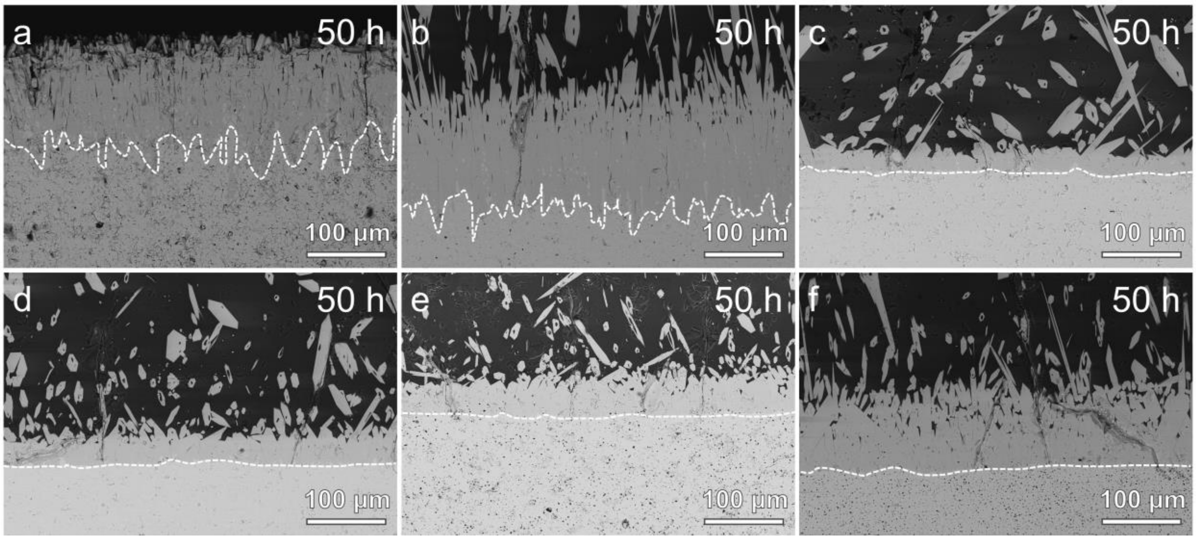

Figure S5.

CMAS post-corroded cross-sections of six samples (100 h). a Lu_2SiO_5, b $(Lu_{1/2}Yb_{1/2})_2SiO_5$, c $(Lu_{1/3}Yb_{1/3}Tm_{1/3})_2SiO_5$, d $(Lu_{1/4}Yb_{1/4}Tm_{1/4}Er_{1/4})_2SiO_5$, e $(Lu_{1/5}Yb_{1/5}Tm_{1/5}Er_{1/5}Ho_{1/5})_2SiO_5$, and f $(Lu_{1/6}Yb_{1/6}Tm_{1/6}Er_{1/6}Ho_{1/6}Y_{1/6})_2SiO_5$.

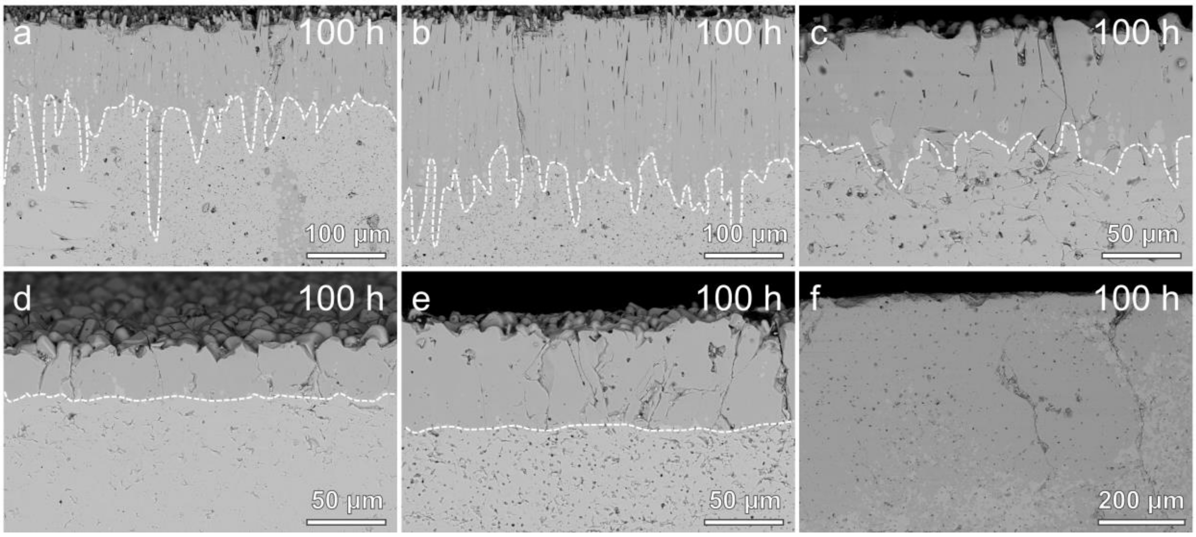

Figure S6.

Typical EBSD datasets of CMAS post-corroded cross-sections of samples (100 h) (image quality maps, phase maps, IPF Y maps, local misorientation distribution images, and pole figure of apatites and monosilicates). a Lu_2SiO_5, b $(Lu_{1/2}Yb_{1/2})_2SiO_5$, c $(Lu_{1/3}Yb_{1/3}Tm_{1/3})_2SiO_5$, d $(Lu_{1/4}Yb_{1/4}Tm_{1/4}Er_{1/4})_2SiO_5$, e $(Lu_{1/5}Yb_{1/5}Tm_{1/5}Er_{1/5}Ho_{1/5})_2SiO_5$, and f $(Lu_{1/6}Yb_{1/6}Tm_{1/6}Er_{1/6}Ho_{1/6}Y_{1/6})_2SiO_5$.

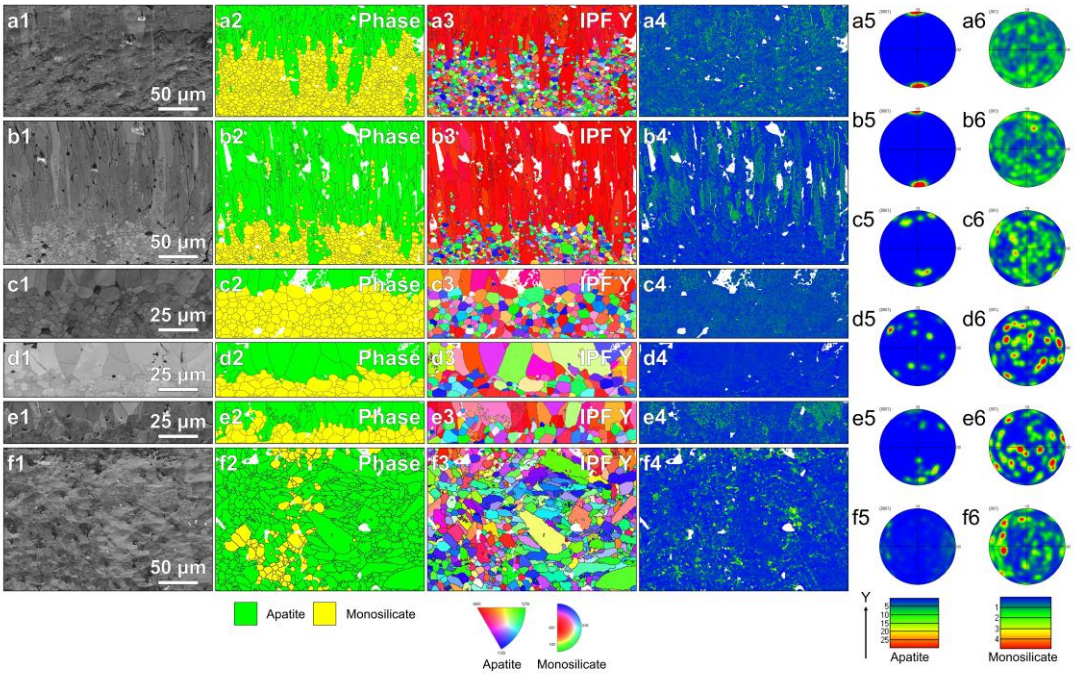

Figure S7.

The strain growth distribution and quantitative analysis of the final apatite products of $(Lu_{1/3}Yb_{1/3}Tm_{1/3})_2SiO_5$. a, b SEM images, c–h The strain growth distribution, and i Quantitative analysis.

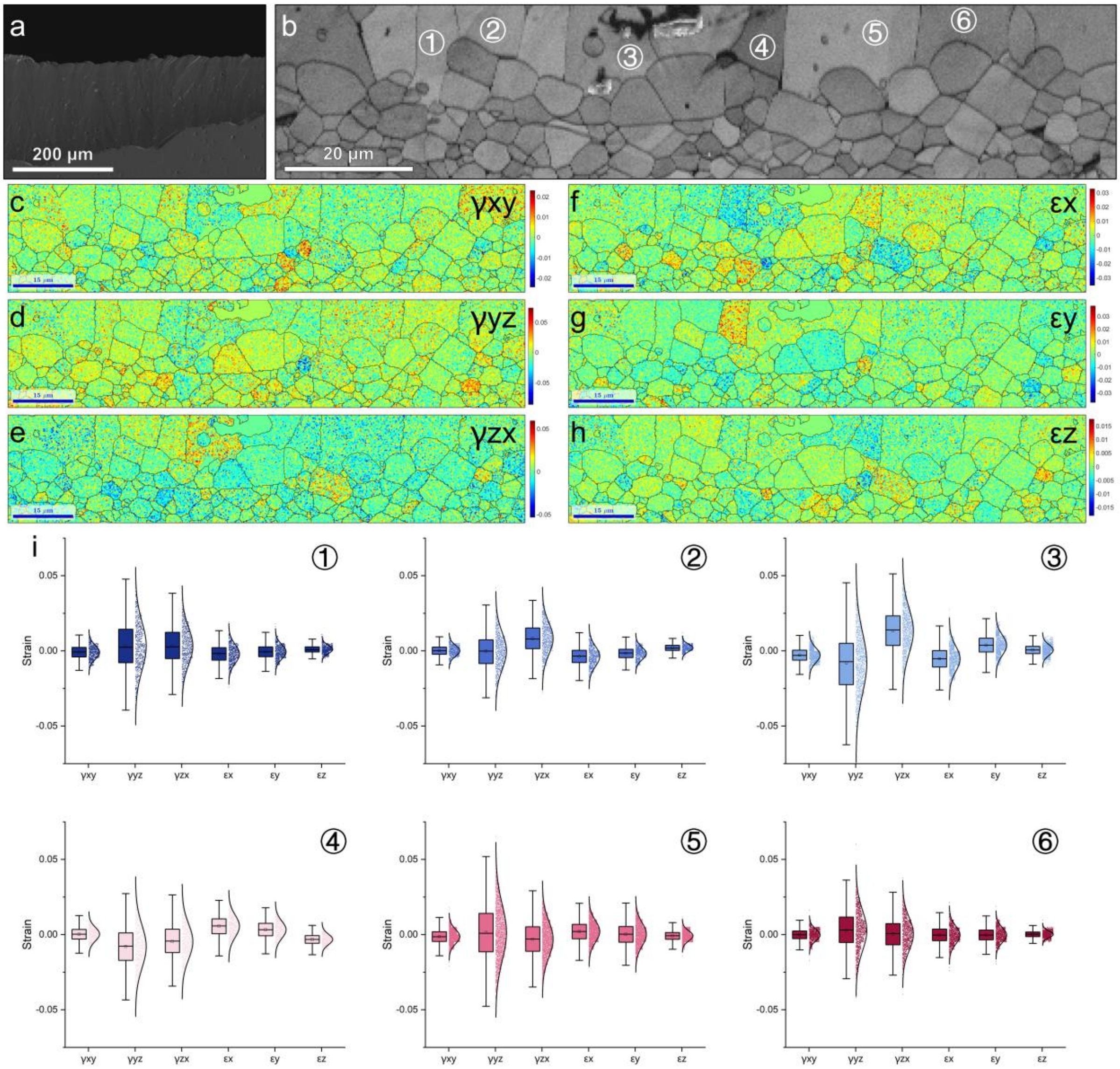

Figure S8.

Rietveld refinement of XRD patterns of six types of apatite products. a $Ca_2Lu_8(SiO_4)_6O_2$, b $Ca_2(Lu_{1/2}Yb_{1/2})_8(SiO_4)_6O_2$, c $Ca_2(Lu_{1/3}Yb_{1/3}Tm_{1/3})_8(SiO_4)_6O_2$, d $Ca_2(Lu_{1/4}Yb_{1/4}Tm_{1/4}Er_{1/4})_8(SiO_4)_6O_2$, e $Ca_2(Lu_{1/5}Yb_{1/5}Tm_{1/5}Er_{1/5}Ho_{1/5})_8(SiO_4)_6O_2$, and f $Ca_2(Lu_{1/6}Yb_{1/6}Tm_{1/6}Er_{1/6}Ho_{1/6}Y_{1/6})_8(SiO_4)_6O_2$.

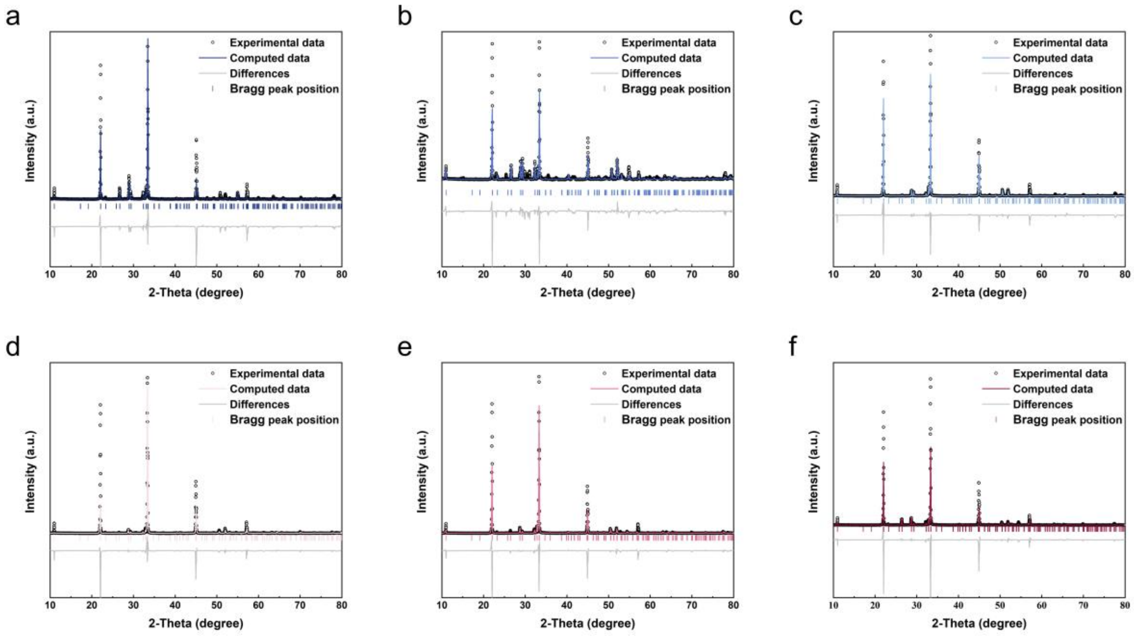

Table S1.

Element compositions of various points presented in Fig. 1b.

EDS/at.%	O	Si	Y	Ho	Er	Tm	Yb	Lu
1	62.50	12.49	3.87	4.10	4.29	4.18	4.36	4.21
2	62.54	12.68	---	4.83	5.00	5.20	4.90	4.86
3	62.56	12.80	---	---	6.09	6.09	6.18	6.27
4	62.55	12.74	---	---	---	8.05	8.36	8.30
5	62.66	13.30	---	---	---	---	11.90	12.14
6	62.70	13.49	---	---	---	---	---	23.81

Table S2.
Element compositions of various points presented in Fig. 4.

EDS/at.%	O	Mg	Al	Si	Ca	Y	Ho	Er	Tm	Yb	Lu	Phase
1	60.85	3.80	5.37	18.08	10.03	---	---	---	---	---	1.88	CMAS
2	62.14	0.19	0.41	15.50	4.58	---	---	---	---	---	17.18	Apatite
3	60.13	6.31	4.72	15.28	8.34	---	---	---	---	---	5.23	Garnet
4	62.78	---	0.56	14.06	0.16	---	---	---	---	---	22.44	monosilicate
5	60.75	4.28	5.36	17.97	9.94	---	---	---	---	0.80	0.90	CMAS
6	61.78	0.74	---	14.41	4.75	---	---	---	---	9.53	8.79	Apatite
7	60.23	6.06	4.16	15.47	8.29	---	---	---	---	2.94	2.86	Garnet
8	62.38	0.74	---	12.74	0.07	---	---	---	---	12.15	11.92	monosilicate
9	60.78	3.84	6.77	17.42	9.68	---	---	---	0.41	0.52	0.57	CMAS
10	61.79	0.52	2.08	14.05	4.56	---	---	---	6.07	5.72	5.21	Apatite
11	62.33	0.60	2.05	12.28	0.05	---	---	---	7.63	7.66	7.42	monosilicate
12	60.80	3.57	7.40	17.15	9.60	---	---	0.25	0.32	0.43	0.48	CMAS
13	61.84	0.37	1.33	14.46	4.87	---	---	4.82	4.40	4.14	3.75	Apatite
14	62.49	0.12	0.92	12.66	0.09	---	---	5.90	5.96	6.02	5.85	monosilicate
15	60.62	4.15	5.99	17.41	10.14	---	0.24	0.30	0.31	0.40	0.43	CMAS
16	61.88	---	---	14.25	4.86	---	4.10	4.07	3.86	3.76	3.22	Apatite
17	62.51	---	---	12.58	0.04	---	5.07	5.06	5.06	4.96	4.72	monosilicate
18	60.72	4.31	6.52	17.57	9.68	0.06	0.17	0.19	0.22	0.26	0.32	CMAS
19	62.08	---	---	15.07	4.67	3.20	3.06	3.11	3.02	2.92	2.85	Apatite
20	62.52	---	---	12.70	0.12	3.59	4.10	4.37	4.31	4.27	4.03	monosilicate

Table S3.

Crystal cell parameters of six types of apatite products as obtained by XRD-Rietveld refinement.

Compounds	a(Å)	c(Å)	γ(°)
$Ca_2Lu_8(SiO_4)_6O_2$	9.2729	6.6709	120.000
$Ca_2(Lu_{1/2}Yb_{1/2})_8(SiO_4)_6O_2$	9.2812	6.6835	120.000
$Ca_2(Lu_{1/3}Yb_{1/3}Tm_{1/3})_8(SiO_4)_6O_2$	9.3067	6.7121	120.000
$Ca_2(Lu_{1/4}Yb_{1/4}Tm_{1/4}Er_{1/4})_8(SiO_4)_6O_2$	9.2933	6.7171	120.000
$Ca_2(Lu_{1/5}Yb_{1/5}Tm_{1/5}Er_{1/5}Ho_{1/5})_8(SiO_4)_6O_2$	9.3024	6.7318	120.000
$Ca_2(Lu_{1/6}Yb_{1/6}Tm_{1/6}Er_{1/6}Ho_{1/6}Y_{1/6})_8(SiO_4)_6O_2$	9.3016	6.7373	120.000
